# Shock

**Published:** 1898-09

**Authors:** 


### Shock.

It is an established fact in surgery that profound and even fatal shock may be caused simply by mental impression. Desault relates a case in which he was about to perform an operation for lithotomy, and in showing the class where he was going to make the incision he merely traced a line with his finger-nail, and the patient, under the impression that a cut was being made, expired from shock.—*N. Y. Med. Times.*

